# Multibody analysis and soft tissue strength refute supersonic dinosaur tail

**DOI:** 10.1038/s41598-022-21633-2

**Published:** 2022-12-08

**Authors:** Simone Conti, Emanuel Tschopp, Octávio Mateus, Andrea Zanoni, Pierangelo Masarati, Giuseppe Sala

**Affiliations:** 1 GeoBioTec, Department of Earth Sciences, NOVA School of Science and Technology, Campus de Caparica, 2829 516 Caparica, Portugal; 2 Department of Aerospace Science and Technology, Politecnico di Milano, via La Masa 34, 20156 Milan, Italy; 3 Universität Hamburg, Martin-Luther-King-Platz 3, 20146 Hamburg, Germany; 4 American Museum of Natural History, Central Park West @ 79th St, New York, NY 10024 USA

**Keywords:** Biomechanics, Palaeontology

## Abstract

Sauropod dinosaurs are well known for their massive sizes and long necks and tails. Among sauropods, flagellicaudatan dinosaurs are characterized by extreme tail elongation, which has led to hypotheses regarding tail function, often compared to a whip. Here, we analyse the dynamics of motion of a 3D model of an apatosaurine flagellicaudatan tail using multibody simulation and quantify the stress-bearing capabilities of the associated soft tissues. Such an elongated and slender structure would allow achieving tip velocities in the order of 30 m/s, or 100 km/h, far slower than the speed of sound, due to the combined effect of friction of the musculature and articulations, as well as aerodynamic drag. The material properties of the skin, tendons, and ligaments also support such evidence, proving that in life, the tail would not have withstood the stresses imposed by travelling at the speed of sound, irrespective of the conjectural ‘popper’, a hypothetical soft tissue structure analogue to the terminal portion of a bullwhip able to surpass the speed of sound.

## Introduction

The extremely elongated tails of diplodocid flagellicaudatan sauropods like *Brontosaurus* have always intrigued researchers and enthusiasts alike^1,2^. Although no complete diplodocid tail has been found so far, the general morphology and the approximate number of elements can be gleaned from overlapping partial findings. These show that the diplodocid tail consisted of approximately 80 caudal vertebrae that gradually decrease in overall size and morphological complexity towards the posterior tip^3^. Approximately 10 large and complex elements form the base, followed by about 40 intermediate elements, and finally by 30 progressively smaller, rod-like vertebrae. This peculiar morphology has inspired many hypotheses to explain tail function. These include (i) acting as a “third leg” during a bipedal standing posture^4^, an interpretation that proved to be questionable based on the anatomy of the caudal vertebrae^5^; (ii) acting as a counterbalance to the long neck^6^; (iii) providing insertion points for the long caudofemoralis muscle^7^; (iv) as a defensive weapon; (v) as a noise-making structure^8^; and (vi) as a tactile device for spatial awareness^9^.

Unlike most other sauropods, diplodocids possessed posterior-most caudal vertebrae that are elongated and rod-like, lack a neural arch, and have biconvex articular surfaces, which allowed for great interelement mobility^2,8,10^. Since the first findings of terminal caudal vertebrae of diplodocid tails, researchers have suggested the possible use of the distal tail as a defensive weapon^2,8,11,12^. This historical hypothesis is supported by the morphological similarities between the anatomical structure of the tail and a bullwhip, with a thick proximal portion and a narrow and light terminal portion, the whiplash^8^. This defensive functional hypothesis has been tested with computer simulations^8,13^, one of which led to the additional hypothesis that the tail was also adapted for noise-making^8^.

The first computer simulation of sauropod tail motion used an apatosaurine tail modelled with 82 segments^13^, each corresponding to a vertebra. This study imposed an initial condition of a coiled tail, simulating its unfolding; and during the simulation, the tail tip could not reach supersonic speed. In a subsequent work based on a different modelling approach, Myhrvold & Currie^8^ approximated the tail morphology in 14 segments, each representing groups of 5–8 vertebrae, and added a hypothetical “popper” at the tail's end—a conjectural soft tissue structure that would render the tail 1 m longer. During the simulations, applying the motion to the first segment at the base of the tail, the tail tip, and in particular the popper, overcame the speed of sound, reaching approximately 560 m/s in standard air.

Here, we apply a novel multi-faceted approach for biomechanical analysis to re-assess diplodocid tail motion and speed. We combine state-of-the-art multibody modelling with simulations of soft tissue resistance to stress, which is a highly promising approach to testing the biomechanical performance of extinct organisms. We created the first multibody model of an apatosaurine diplodocid tail, and simulate, for the first time, soft tissue resistance to the stress imposed on the skin, tendons, and ligaments, when moving at the speed of sound.

The internal soft-tissue morphology of the sauropod tail is unknown, despite several specimens with preserved skin impressions^14,15^. In vertebrates, the vertebral column is held together by ligaments connecting the neural spines and others connecting the vertebral centra; in humans, these are called the anterior longitudinal ligament and the posterior longitudinal ligament, and together they enfold the vertebral centra^16^. Considering that the terminal portion of the diplodocid tail lacks neural spines^2^, it is reasonable to hypothesize that only the longitudinal ligaments occurred in distal diplodocid tails, forming an envelope connecting the vertebral centra. In this study, different materials have been considered as representatives of the soft tissue structure of the diplodocid tail.

The mechanical properties of soft tissues, as for any viscoelastic material, are dependent on the strain rate, with the tissues changing from a viscoelastic behaviour at low strain rates to stiffer and more brittle behaviour at higher strain rates, thereby reducing the maximum deformation bearable before breaking^17–20^. Skin becomes almost completely brittle at high strain rates, with an Ultimate Tensile Stress (UTS; this corresponds to the maximum stress applicable on the material beyond which the material would break) ranging between 17 and 26 MPa^18–22^. Compared to tendons and ligaments, skin fails first because it has the lowest UTS of these three tissue types at any applied strain rate. Mean values of UTS for tendons range from 46,64 MPa to 69,33 MPa; moreover, tendons are stiffer than skin at similar strain rates^23^. Ligaments maintain the highest UTS, with values ranging between 50 and 150 MPa^24^. Skin is a complex connective organ with several layers. The absolute and relative thickness of these layers varies along the body, and across taxa; the thickness of the domestic pig epidermis ranges between 30 μm and 140 μm, that of humans between 50 and 120 μm^25^, and crocodile skin is composed of an epidermis that is 30–150 μm thick, sitting on top of a variably thick dermis, which also includes bony osteoderms (250–500 μm)^26^. Because no specimen of fossilized skin associated with the distal-most portion of diplodocid tails preserves its thickness, we base our simulations on the thickness of crocodile skin. However, given the lack of analyses of the mechanical properties of crocodile skin, we must use the mechanical properties of mammal skins. Despite cow and kangaroo hide have better performance than pig and human skins, they lack any data on UTS at high strain rate, for this reason the latter were preferred. In this regard leather, tendons and ligaments have been considered in this study, testing a wider array of materials and range of UTS values.

The anisotropic behaviour, and thus the mechanical properties, of the skin is dictated by three main factors, which all relate to the dermis^20^: (i) the alignment of the so-called Langer’s lines, topological lines parallel to the orientation of collagen and muscular fibres; (ii) the structure composed by the alignment of collagen fibres, which evolves with the deformation; and (iii) the strain rate^17–22,27–29^. Several studies focused on the mechanical properties of compressive^22,29^ and tensile^19,29^ behaviour of the skin, referring to the UTS in both cases. However, the skin has different mechanical properties in tension and compression^29^. Given that it is mainly the centrifugal force that acts on the tail when moving at maximum speed, we consider only the values derived from tensile tests. We also assume that the Langer’s lines of sauropod tails were aligned with the craniocaudal axis, parallel to the stress direction, which coincides with the highest mechanical performances of the skin^18,20,21,29^.

Tendons and ligaments are dense fibrous connective tissues, with the former connecting muscles and bones, whereas the latter directly connects bones, adding stability to the skeleton. Like skin, tendons and ligaments possess collagen fibres that confer tensile strength^24^. The greater tensile strength of tendons and ligaments compared to that of the skin is given by the greater percentage of collagen present in these tissues (skin 56–70%; tendons 70–80%; ligaments 75–85%)^24^. The collagen fibres are organized in a wavy pattern, which is embedded in a gel matrix and is generally oriented along the main axis of stress experienced by the tendon or ligament. Tendons have a similar structure to ligaments but with the collagen fibres organized in packets. This arrangement, as happens to the skin, evolves with the deformation of the tissue^24^. At slow strain rates, the arrangement of the collagen fibres determines the typical behaviour of the material, which is composed of three phases: (i) at first the material is ductile, deforming according to the resistance given by the gel matrix; (ii) as the applied stress increases, the increase in deformation follows a linear pattern, while the collagen fibres align to the direction of the stress; (iii) during the third phase, the aligned fibres begin to slide within the gel matrix, altering their relative position, deforming the material until failure^24^. This typical pattern changes with the age of the tissue, reducing the first and second behaviours and increasing its stiffness and UTS as the tissue matures^24^.

## Methods

### Apatosaurine model

#### Model construction

Tail morphology varies in diplodocids, with diplodocines having generally more elongated vertebral centra throughout the tail compared to apatosaurines^10,30,31^. However, because the most complete diplodocids tails are known from apatosaurines^2^, and because the previous models were based on apatosaurine morphology, we also restrict our multibody analysis on this taxon.

Our tail model is inspired by the one used in Myhrvold and Currie^8^ cross-checking the measurements with five specimens generally considered being apatosaurines (CM 3018, CM 3378, AMNH FARB 222, FMNH P25112, UW 15556; taxonomy taken from the Morrison Formation Sauropod Consensus^31^). It combines the properties of both models proposed previously. A single rigid body represents every single vertebra, as in Gertsch^13^, because dividing the tail into rigid segments, as in Myhrvold and Currie^8^, oversimplifies the model, greatly reducing the computational time at expense of the accuracy of the results. As in Myhrvold and Currie^8^, the vertebrae were modelled simplifying their geometry as cylinders and considering them as perfectly rigid bodies. We applied the measures and proportions of their model^8^ and extrapolated the respective data concerning the limitation of rotation between each rigid body.

The masses of the elements representing the vertebrae were considered centroids in the centre of mass, for a total of 82 centroids, dividing the whole model into 14 sections of multiple equal elements (similar to Myhrvold and Currie). The position of the joints has been set considering the shape of the elements and taking into account the dimensions of the cartilaginous intervertebral discs, setting their thickness at 10% of the length of the relative element^32^.The resulting total length of the tail is 12.44 m, with a total weight of 1446.16 kg (Table 1, Supp Mat). The centre of mass of the whole model is located between the fifth and the sixth caudal vertebra.

The previous studies only analysed caudal vertebrae, allowing the first element to achieve a greater arc at the base than what would have been possible when articulated with the sacrum, enhancing its performance. We added another element at the base of the model (i. e. the sacrum), which greatly affected the results, substantially limiting the maximum speed achievable. Omitting these parameters and subdividing the tail into rigid segments may be part of the reason why Myhrvold and Currie found the popper able to overcome the speed of sound.

Although there is no evidence in the fossil record of the presence of a soft-tissue-only popper, Myhrvold and Currie^8^ included in their model such a soft tissue keratinous feature, divided into three segments of 0.33 m length each and measuring the velocity achieved at the tip of this structure. We did not include a hypothetical soft-tissue popper in the model, because its presence would have affected the simulation by acting as an air brake, increasing the air drag of the model, and thereby slowing the movement. Instead, we tested the impact of different popper morphologies when travelling at the speed of sound on soft tissue resistance (see below).

The mathematical model of the tail was created using MBDyn (http://mbdyn.org/), a free general-purpose multibody dynamics analysis software developed by the Department of Aerospace Science and Technology (Politecnico di Milano, Italy)^33^. The software has been used for biomechanics^34,35^, but is here applied to paleontological data for the first time. In the previous simulations, Gertsch^13^ imposed the model to be coiled and applied a constant radial acceleration to all the rigid bodies, measuring the top velocity when the tail was completely stretched, while Myhrvold and Currie^8^ applied torque for 0.2 s at the base of the tail, followed by a counter-torque; to create and propagate the wave down the model.

**Figure 1:**
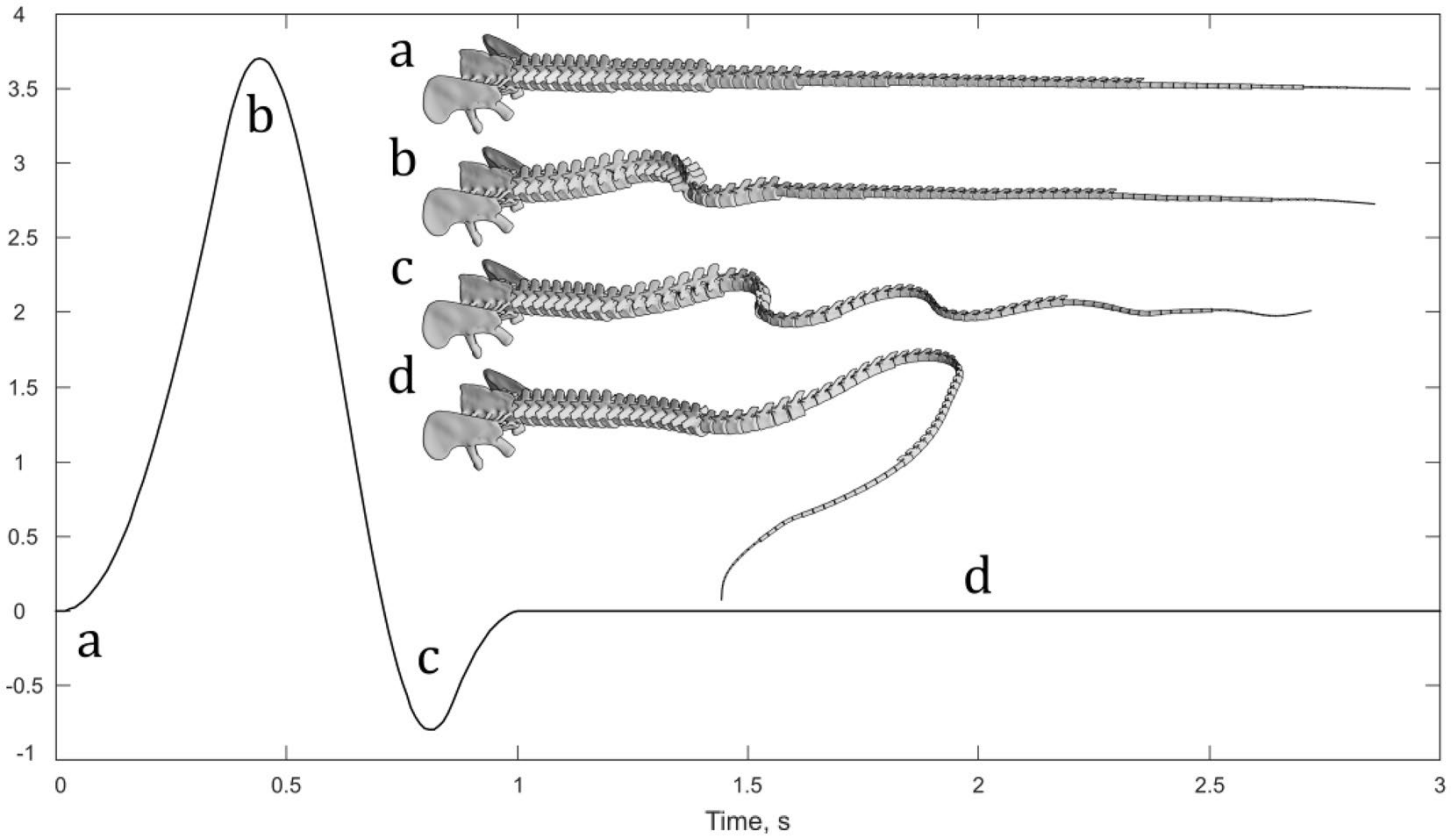
The degrees of rotation with respect to the X-axis imposed with the motion prescribed to the first eight vertebrae. (**a**) Position of the caudal centra at the beginning of the simulation. (**b**) Position of the caudal centra at the end of the first cosine input. (**c**) Position of the caudal centra at end of the second cosine input. (**d**) Position of the caudal centra at 2 s in the simulation, when the first eight centra are stable.

In our model, each element is connected with the previous one by a revolute joint, with no translational degree of freedom allowed and only rotational movement degree of freedom on one axis, around the perpendicular (Y) axis, which corresponds to the dorsoventral axis. The motion is prescribed to the first eight elements, with a function describing their relative rotation as a cosine function of 5°63’ of amplitude and a frequency of 1 Hz for 0.25 s, after which a second cosine function is applied in the opposite direction with 5°63’ of amplitude, a frequency of 2 Hz for a total single cosine cycle (Fig. 1). This movement can generate a wave and accelerate the centre of mass of the whole model to a velocity of around 1–2 m/s as in previous studies^8,13^.

#### Constraints on the model

Each element of the model represents one caudal vertebra, plus a fixed element (i. e. the sacrum) to constrain the model in space. The elements are connected by revolute joints, to allow the rotation only in the plane perpendicular to the dorsoventral axis. The rotation is influenced by a spring superimposed over the joint, to confer stiffness and damping to the articulation, simulating the contraction of the soft tissue; with values proportional to the dimensions of the element (Table 1, Supp Mat), and a coefficient of 5 × 10^6^ N⋅m/rad for stiffness and 1 × 10^6^ N⋅m⋅s/rad for damping. The rotation is limited by the use of a continuous contact formulation that applies a reaction force upon reaching the maximum angle imposed on the model (Table 1, Supp Mat) using a form of the model originally proposed by Hunt and Crossley^36^ and subsequently enhanced by Flores et al.^37^ to provide the desired restitution rate based on the actual velocity at the contact. The model was further improved by adding the drag resulting from interaction with the air, with the approximative formula

$${drag}_{air} = \frac{1}{2}\rho {{v}_{\perp }}^{2} S {C}_{D}$$

considering the density of air (ρ) at sea level at 15 °C, equal to 1.225 kg/m^3^; the component of velocity (*v*_⊥_) perpendicular to the movement; the section (*S*) of the element given by the length of the segment multiplied by its average diameter, assuming cylindrical segments with the corresponding drag coefficient (*C*_*D*_) of 0.5.

We imposed a rotation of maximally 5.63° within 0.25 s to the first eight vertebrae and a subsequent counter-rotation to complete the movement. Following the tail sections in the model, this maximum allowable angle was increased step-wise to reach 9° between caudal vertebrae 42 and 43 and more distal joints. These chosen angles of rotation correspond to morphological constraints, with a higher angle leading vertebrae to contact each other. Because of technical limitations, the intervertebral joints were allowed to accommodate a wider rotation during the simulation than what we deemed likely based on their osteology. Enforcing a more limited angle of rotation leads to non-convergent results in the simulation, and to its abortion. The non-converging results of the simulations are assumed as the incapability of the model to maintain the geometry, thus would represent a disarticulation of the model, a phenomenon not feasible for the animal.

#### Soft tissues strength

The allowable ultimate stress (UTS) of the materials considered in this analysis was valued as the UTS of brittle materials and yield stress of ductile materials. The ultimate force bearable by the soft tissues must be equal or greater than the centrifugal force applied by the rotation, otherwise, the tail would fail. Hooke’s law states that, at given force, stress is inversely proportional to the area it is acting upon $\sigma = F/A$ where *F* is the applied force and *A* is the area on which *F* is applied. The area in question would refer to the cross section of soft tissue present in a section of the tail and can be extrapolated assuming the vertebra’s shape as cylindrical. Considering a general density (ρ) of 1000 kg/m^3^^8^ at the terminal portion of the tail, and considering just the last vertebra of the tail having a mass (*m*) of 0.0542 kg and length of 60 mm as in the model of Myhrvold and Currie^3^, the radius of the cylinder results being 17 mm. The distance from the centre of articulation of the last vertebra and its centre of mass is estimated to be 40 mm, considering half the length of the last element and adding the space for the articular joint and the convex articular facet. To estimate the area necessary to withstand the stress imposed by the last vertebra travelling at 340 m/s two geometric hypotheses have been tested per each material considered: a disc, aligned with the centre of mass of the vertebra; and a circular crown, which would envelop the vertebra.

#### Popper

The presence of soft tissue structures at the end of the sauropod tail, analogous to the popper of the whip cannot be completely ruled out just in the absence of fossil findings. Such a structure can be hypothesized to be composed of different materials, such as skin, tendon, keratin^8^, and maybe keratinous filaments as those present in other dinosaur taxa^38^. We tested how three different morphologies of a popper would sustain the stress of moving at the speed of sound. The first morphology corresponds to the model proposed by Myhrvold and Currie^8^, with a popper divided into three segments, each of 0.33 m in length, with respective masses of 0.022, 0.015, and 0.009 kg^3^. The second morphology considers a popper composed of keratinous filaments connected to different posterior vertebrae, braided to create an empty mesh. The third morphology is similar to a flail, with an additional mass composed of soft tissues, which is connected to the end of the tail by other soft tissues.

### Ethic declaration

No animal or animal tissue samples were used in this study.

## Results

**Figure 2:**
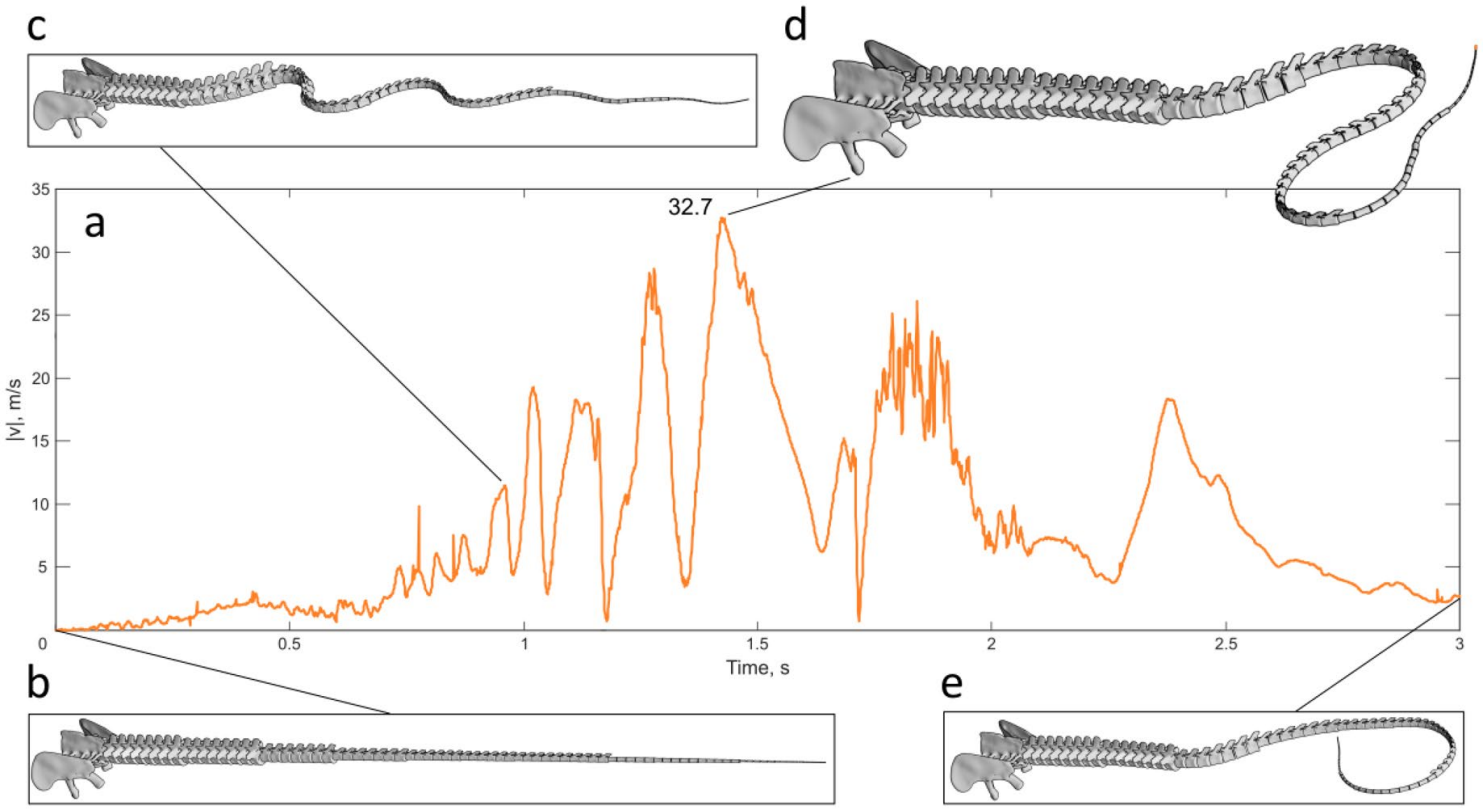
The velocity of the last vertebra during the simulation. (**a**) the velocity of the centrum of the last tail vertebra in m/s in the time of the simulation including the effects of air drags. (**b**) Position of the caudal centra at the beginning of the simulation, (**c**) position of the caudal centra at 1 s from the beginning of the simulation. (**d**) Position of the caudal centra at the moment the last centrum reaches the top speed (32.7 m/s). (**e**) Position of the caudal centra at the end of the simulation.

The imposed movement at the tail base generates a wave that culminates in a rotational movement of the last elements, creating a loop—similar to the behaviour of a whip. As the wave reaches the last elements and unrolls the tail, its velocity increases due to the decrease of mass and tail cross-section. However, the addition of an element simulating the articulation of the tail to the sacrum limits the arc of rotation of the first caudal vertebra, which in turn led to lower maximum velocities of the tail. Adding air drag to the model further reduced the maximum speed achieved, which corresponds to 32.7 m/s at 1.42 s into the simulation (Fig. 2), which is more than 10 times slower than the speed of sound in standard air.

On top of estimating the maximum speed using multibody simulations, we simulated the thickness of skin, tendons, and ligaments that would be required to resist breaking if the tail would travel at the speed of sound (Table 1). We base our simulations on the distal-most vertebra, because that is where the multibody analysis recovered the highest velocities. For each material, we considered different hypothetical anatomies: (i) a cylinder between the articular surfaces of the last two caudal vertebrae, (ii) an envelope connecting the last two vertebrae around a 10-mm-thick core, (iii) an envelope around a 20-mm-thick core, and (iv) an envelope around the whole vertebra with an internal radius of 17 mm. Not all hypothesised structures are compatible with the material considered, yet, no material in any hypothesised condition can prevent the rupture of the tail travelling at 340 m/s (see Table 2 Supp Mat).

**Table 1:** The thickness in cm of the different materials needed to sustain the speed of sound (340 m/s) in two of the hypothesized conditions: considering a cylinder of material and the annulus external to the vertebra.

Material	Skin 17 MPa	Skin 26 MPa	Leather 40 MPa	Tendon 45 MPa	Ligament 50 MPa	Ligament 60 MPa	Ligament 80 MPa	Ligament 150 MPa
Hypothesis radius cylinder (cm)	5.4	4.4	3.5	3.3	3.2	2.9	2.5	1.8
Hypothesis radius external annulus (cm)	4.0	3.0	2.2	2.0	1.9	1.6	1.3	0.8

**Figure 3:**
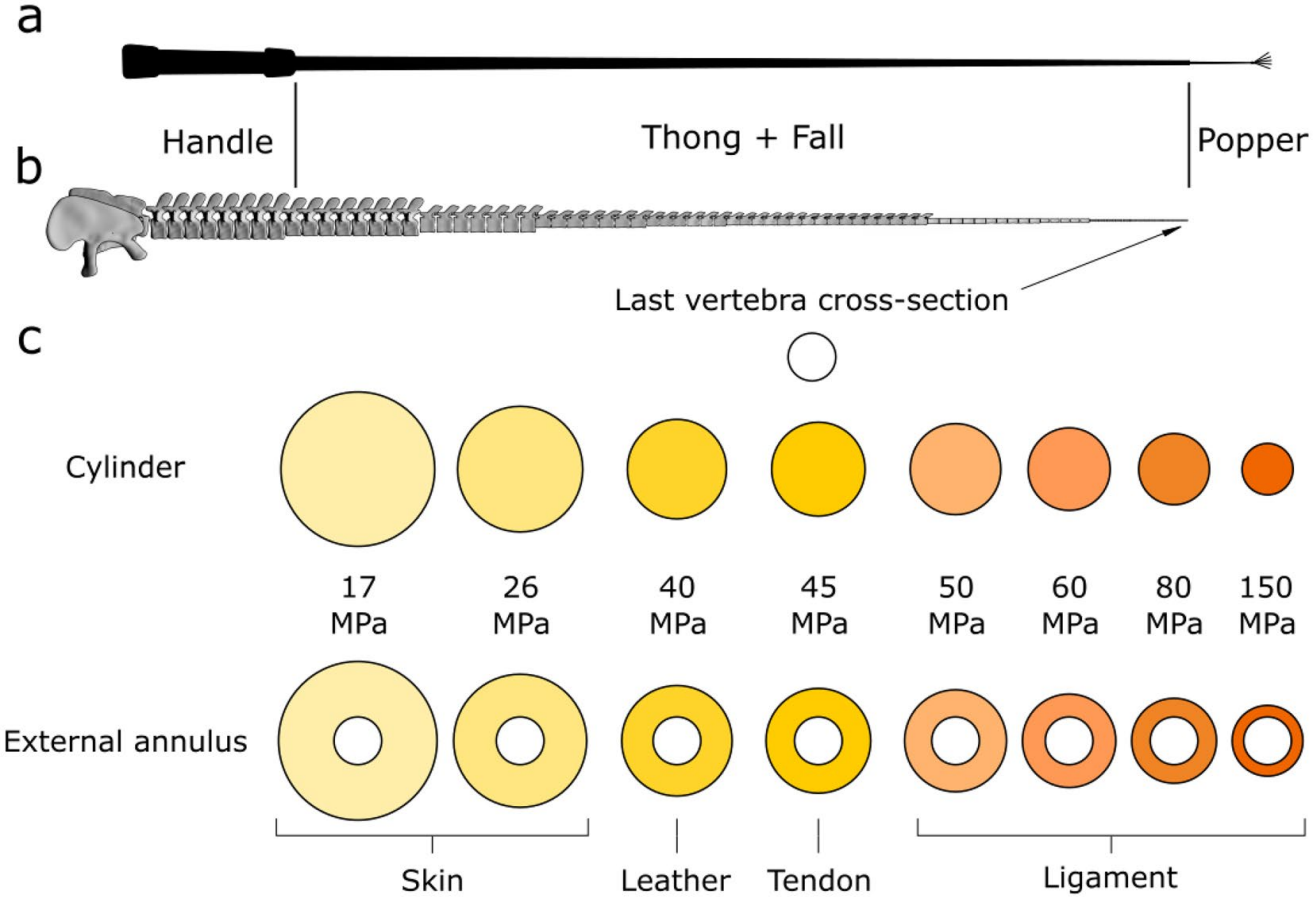
Anatomy of a bullwhip compared to the model and graphical representation of the soft tissue surface needed to bear the supersonic stresses compared to the hypothesized size of the final vertebral element. (**a**) Drawing of a bullwhip. (**b**) Model of sauropod tail in lateral view. (**c**) Graphical representation of the cross-section of the last vertebral element, compared to the cross-sections of soft tissues needed to bear the supersonic stresses. The first row represents the size of the soft tissue as cylindrical structure. The second row represents the size of soft tissues as annulus external to the hypothesized vertebral element.

When the motion pattern reaches maximum speed, the centrifugal force becomes the predominant factor of the stresses acting on the soft tissues of the tail. These stresses would be distributed across the soft tissue, concentrating on the parts where the soft tissues are thinnest, i.e., around the vertebral centra or the vertebral joints. Assuming that the soft tissue of the tail would be composed exclusively of skin and implementing mechanical properties like those of leather, the cross-section of the soft-tissue cylinder required to prevent the failure of the tail would have to have a radius of 35 mm, more than twice the size of the whole vertebra, which is composed of cylindrical elements of 17 mm radius (Table 1). Living tissues have considerably worse performances than leather, thus requiring greater surfaces of the cross-section to distribute the stress. If the stresses would be supported by tendons only, it would require a tendon of 33 mm radius connecting the last two vertebrae to withstand the speed of sound, assuming a UTS for tendons of 45 MPa^23^. Despite having a UTS of up to 150 MPa, also ligaments would not withstand the stress imposed by the tail travelling at the speed of sound, requiring a bigger surface of the cross-section than available (Table 1). In all cases, if we increased soft tissue thickness to sustain greater stresses, the thicker tissue would increase the mass, which would, in turn, further increase the applied centrifugal force, again requiring an additional increase of soft tissue thickness. Extending these calculations from the distal-most articulation to more proximal vertebrae would also increase the mass that would have to be supported by the soft tissue because the mass set in motion would be the combined mass of all vertebrae distal to the articulation in question. Given the relatively constant morphology of the last 30 or more vertebrae^2^, which are all slender and cylindrical, and with similar proportions, there is no evidence for a variable thickness of the soft tissue associated with these posterior-most caudal vertebrae, as would be required to resist stresses imposed by travelling at speeds comparable to that of sound. Thus, our results show that the cross-sectional area of any soft tissue type required to withstand the stress of reaching the speed of sound exceeds the hypothesized dimensions of the tail itself; even attributing the best-performing parameters obtained by any given soft tissue material (Fig. 3).

The previous suggestion of supersonic diplodocid tails was based on their similarity with a bullwhip^8^. A bullwhip is composed of several parts: a handle; a lash with a decreasing number of components; and the tip of the whiplash, commonly called the popper, which is a single piece of material behind a knot, frayed out to create a brush or a tuft^39^. The morphology of supersonic whips, and the popper, in particular, is optimized to produce the typical cracking sound when surpassing the speed of sound (340 m/s)^39–41^. Additional knots can be added to achieve the cracking sound more easily because they increase the mass and thus the kinetic energy at the tip of the whip^39,41^. Cracking a whip leads to extreme tension in the lash provoking heavy wearing and tearing of the tuft, which requires continuous trimming^39^. There is no evidence in the fossil record of any such structure as the tuft or an increased mass in the terminal elements of sauropod tails, and the morphology of the preserved terminal caudal vertebrae shows a slender and elongated cylindrical shape. A cylindrical shape is not optimal to increase mass, instead, it rather increases the surface, consequently increasing the air drag acting on it. However, because the popper is of fundamental importance in creating the sonic boom in whips, and because it was mostly this structure that surpassed the speed of sound in the simulation of Myhrvold and Currie^8^, we tested if a popper would have withstood a supersonic movement of the tail. The soft tissue estimates revealed that none of the three hypothesized structures would be able to accommodate the motion at supersonic speed, leading to the failure of the popper or the distal-most tail where the popper would be attached (see Supplementary Materials, Table 3).

The first morphology we tested has been hypothesised to be composed of only a single material, to simplify the calculation. In the simulation resulting from the study of Myhrvold and Currie^8^, the popper surpassed the speed of sound (340 m/s) for about 0.008 s, reaching 560 m/s at around 0.5–0.6 s into the simulation, changing speed from ≅ 40 to ≅ 500 m/s in ≅ 0.025 s, with an average acceleration of 18,400 m/s^2^, which would correspond to 1875 g. It has been considered as composed of skin, which would not resist the stress applied, and keratin. The density of the latter varies between 1283 and 1355 kg/m^3^^42^; both values have been considered in the analysis. The UTS for keratin has been valued at 240 MPa, obtained from tests performed on the calamus of a goose feather^43^. Even if the popper would have been able to sustain the movement at supersonic speed, the increased mass at the end of the tail would increase the stress applied to the vertebrae, leading the tail to fail. It should also be considered that if the tip of the tail would reach the speed of sound, the popper would increase its velocity even further, due to the decreased size acting as the popper of a whip, and the increased arm between the centre of mass of the popper and the centre of rotation posed at the end of the last vertebra. The second morphology, of a popper composed of braided keratinous filaments, would not be feasible because to sustain a certain hypothesized mass of keratinous filaments would require a greater surface, which would lead to an increased mass of the popper, thus increased stress applied, leading the popper to fail. As in the previous morphological scenarios, the third morphology is not feasible due to the fact that it is an increased mass acting on the last vertebra, and the soft tissue connection would deform under stress, leading to an increased distance between the centre of rotation and the centre of mass. This stretched popper would also suffer from the necking phenomenon, not being constrained by any means, which would decrease the surface on which the forces are concentrated, increasing the stress applied and leading, either the tail or the popper, to failure. Each morphology has highlighted different lines of evidence that come to the conclusion that the tail, even with a soft tissue popper, would not sustain a movement at supersonic speed.

Increasing the mass would increase the force applied to the tail vertebrae. The addition of a soft tissue popper at the end of the tail would represent an increase in the mass moving. An increased mass represents an augmented force acting on the soft tissues connecting the tail vertebrae, which would augment the stress applied, leading to tail failure. To increase the resistance to the stress applied, the cross-section of the soft tissues must be enlarged, but this would lead to an increased mass and an increase of the lateral surface. An increased lateral surface would augment the air drag acting against the movement, slowing the tail.

The second morphology of the popper, with braided keratinous filaments, would decrease the stress applied to every single vertebra, anyway, the stresses will be concentrated in the vertebra where the first filaments of the popper will start, since all the subsequent vertebrae, and relative filaments, are connected to this element. An envelope of keratinous filaments would increase the stiffness of the portion of the tail, making it act as a single body, increasing the stresses where it is attached to the tail. The empty braided structure would also act as an airbrake, due to having a small mass compared to the surface the braided structure would create, slowing the tail movement. The surface of the braided popper would also have a higher air drag coefficient, due to its rugose surface, which would create turbulence on its surface, increasing the air drag. Having a tuft of unbraided keratinous filaments would only increase the air drag acting on the tail, slowing the tail even further.

A soft tissue popper would be composed of viscoelastic materials, which would deform under stress increasing the centrifugal force acting on it and increasing the stress applied. A popper with increased mass at the end of it attached with soft tissues to the last vertebra would fail due to the deformation of the soft tissues under stress. A viscoelastic material would be deformed by the stress applied, this deformation would increase the arm, the distance, at which the popper is moving with respect to the tail. The increased arm would augment the tangent velocity, leading to an increased force applied.

## Discussion

### Limitations of 3D model and multibody simulation do not impact our results

Models are always affected by limitations, as their use itself is a simplification of reality. In palaeontology the lack of data on actual specimens (in particular regarding their soft tissue anatomy) forces us to use modern analogies, which further increases the errors inherent to each such study. Our model is characterised by adding an unmovable sacrum, affecting the results of the simulation. The lateral oscillations of the hip would potentially amplify the movement and probably increase the maximum speed achievable. In any case, however, even if the hip would greatly increase the movement of the tail, our estimate of soft tissue resistance would not support the supersonic movement of dinosaur tails.

### Scaling is an important factor

Diplodocid tails are often compared to whips, mostly due to their morphological similarity. Myhrvold and Currie even showed that diplodocid tails have the same proportions in diameters as a bullwhip^8^, yet the actual dimensions are the critical weakness in this comparison. An object fails once the material composing the object reaches its maximum deformation at the outer edge of the object^27^. At high speeds, sauropod tail would fail, while a whip will hold simply for their difference in absolute size: having a larger diameter results in a greater deformation at the outer edge, because the edge is further away from the neutral axis during the flexion caused by the movement^27^.

### Functional and behavioural implications

Even though a use of apatosaurine tails to create a supersonic boom does not seem feasible, our results support the potential use of the tail as a defensive weapon, or for intraspecific combat. Even using the most conservative results of the multi-body simulation, the tail tip would achieve a maximum speed of ≅ 30 m/s. This datum can be used to estimate the force, thus the pressure, of a blow delivered to another body. Considering a generic section of the final portion of the tail and imposing the speed of 30 m/s the kinetic energy can be calculated, that would be transmitted on impact. The work will be equal to the force multiplied by the deformation. Assuming a deformation of 1 cm, as compression of the soft tissues, around a third of the diameter of the terminal element, we can calculate the force of the impact, which, divided by the surface of the impacting tail, gives us the pressure applied. The pressure applied by a terminal section of the tail travelling at 30 m/s would be equal to the pressure applied by a golf ball travelling at 88 m/s (≅ 315 km/h) or a volleyball travelling at 57 m/s (≅ 205 km/h). Such pressure would not be able to break bones or lacerate skins but would deliver a sensible blow to the external body, as well as to the tail itself. Trying to create behavioural hypotheses is conjectural, there is little evidence that can help us speculate the tail use. Based on the pressure applied by such an impact, it is clear that the use of the tail as a defensive weapon is plausible and may have the capability to produce enough pain without breaking bones or lacerating skin, which is fundamental given that the same forces would be applied to the tail itself. However, it remains speculative if this use as a weapon was mainly against predators^2,12^, during intraspecific combat^44^, or both. Similarly also a use of the tail to maintain herd cohesion^9^ remains possible but lacks any supporting evidence.

## Conclusions

Based on our analyses, the actual speed diplodocid tails could reach was considerably lower than previously reported, and soft tissue structures connecting the distal tail vertebrae would not be capable of resisting the tensile forces implied by travelling at the speed of sound. Diplodocid tails were thus stiffer than previously thought, with an important role played by the tendons and musculature to avoid disarticulation of the vertebrae once the tail is set in motion. The hypothesis of a sauropod supersonic tail is unsupported by the evidence obtained by the computer simulation and the estimates on the stress-bearing of soft tissues. The limitation at the base of the tail imposed with the articulation with the sacrum and the action of air drag reduces the maximum speed achievable. A soft tissue popper would not withstand the high stresses imposed by the motion at the speed of sound since the increased mass would lead to failure of the tail, or the increased air drag would further reduce the tail speed. The main lines of evidence against the presence of a supersonic popper relate to the increased mass at the end of the tail, which would increase the applied stresses to the soft tissue connecting the last vertebrae; an additional structure having a high surface/weight ratio would act as an air-brake, slowing the movement and preventing the reach of supersonic speed; a soft tissue popper would suffer the deformation due to the high stress applied, and the deformation would lead to a decreased cross-section, increasing the pressure applied, thus causing the structure to fail. The use of the tail as a defensive weapon is not questioned and remains a plausible explanation for the morphology of the terminal portion of the diplodocid tail. These results highlight the utility of our novel approach to biomechanical analysis in palaeontology, combining evidence from modelling hard and soft tissue structures based on contemporary multibody simulations and application of Hooke's law, for the first time in a palaeontological context. Hooke’s law based on simple geometry and fundaments of continuum mechanics theory is commonly applied in engineering. Its simplicity and effectiveness make it viable for future paleontological and biological studies.

## Supplementary Information

Supplementary Table 1.

Supplementary Table 2.

Supplementary Table 3.

Supplementary Video.

## Data Availability

All data generated or analysed during this study are included in this published article, and its supplementary information files.
